# Clinical Profile and Risk Factors for Cardiac Death in Pediatric Patients With Primary Dilated Cardiomyopathy at a Tertiary Medical Center in China

**DOI:** 10.3389/fped.2022.833434

**Published:** 2022-04-28

**Authors:** Yan Wang, Bo Han, Youfei Fan, Yingchun Yi, Jianli Lv, Jing Wang, Xiaofei Yang, Diandong Jiang, Lijian Zhao, Jianjun Zhang, Hui Yuan

**Affiliations:** Department of Pediatrics, Shandong Provincial Hospital Affiliated to Shandong First Medical University, Jinan, China

**Keywords:** dilated cardiomyopathy, heart failure, pediatric patient, mortality, risk factor

## Abstract

**Aim:**

We sought to identify the clinical characteristics and risk factors for cardiac mortality in pediatric patients with primary dilated cardiomyopathy (DCM) in China.

**Methods:**

A total of 138 pediatric patients who were consecutively diagnosed with primary DCM from January 2011 to December 2020 were included. We assessed patients’ clinical symptoms and performed laboratory examinations, electrocardiography, and echocardiography.

**Results:**

Of these patients, 79 (57%) had severe systolic dysfunction (left ventricular ejection fraction of < 30%), 79 (57.2%) developed DCM before 12 months of age, 62 (45%) were male, 121 (87.7%) presented with advanced heart failure (cardiac functional class III/IV), and 54 (39.1%) presented with arrhythmia. At a median follow-up of 12 months, the overall cardiac mortality rate was 33%, and 40 of 46 deaths occurred within 6 months following DCM diagnosis. A multivariate Cox regression analysis identified several independent cardiac death predictors, including an age of 12 months to 5 years [hazard ratio (HR) 2.799; 95% confidence interval (CI) 1.160–6.758; *P* = 0.022] or 10–15 years (HR 3.617; 95% CI 1.336–9.788; *P* = 0.011) at diagnosis, an elevated serum alanine aminotransferase (ALT) concentration (≥ 51.5 U/L) (HR 2.219; 95% CI 1.06–4.574; *P* = 0.031), and use of mechanical ventilation (HR 4.223; 95% CI 1.763–10.114; *P* = 0.001).

**Conclusion:**

The mortality rate of primary DCM without transplantation is high. Age, an elevated serum ALT concentration, and the need for mechanical ventilation predict mortality in patients with primary DCM, providing new insights into DCM risk stratification.

## Introduction

Dilated cardiomyopathy (DCM), the most prevalent type of cardiomyopathy and a common cause of heart failure in children (1, 2), is a serious condition characterized by left ventricular (LV) dilatation and systolic dysfunction with an incidence of approximately 0.53 cases per 100,000 per year in North American children (3). Nearly 40% of children with DCM undergo heart transplantation or die within 2 years of diagnosis (1). Although effective treatments have been developed in recent decades, DCM is still a life−threatening event in many cases (4, 5).

The DCM survival rate is approximate 69–72% at 1 year and 54–63% at 5 years post-diagnosis (1, 6). Although previous studies have investigated the incidence, risk factors, and outcomes of DCM, the predictive effects of these factors differ between studies (7). The clinical course of DCM can vary and is not always unanimous with LV ejection fraction (LVEF) and symptoms (8).

We had access to a large cohort of Chinese pediatric patients who were not due to undergo heart transplantation because of financial constraints or other reasons and who were thus undergoing guideline-directed medical therapy and mechanical ventilation. We sought to identify the clinical characteristics of these patients with primary DCM and the predictors of cardiac death.

## Patients and Methods

### Patients

A total of 138 patients who were consecutively admitted to the Department of Pediatrics at Shandong Provincial Hospital between January 1, 2011, and December 31, 2020, were recruited for this retrospective study. The initial evaluation was the diagnosis with primary DCM at the time of the first clinical assessment at the hospital. Diagnosis and management were performed by at least two pediatric cardiologists. More recent evaluations were performed at the outpatient clinic or by telephone. The diagnosis of DCM was based on echocardiographic evidence of LV dilatation [i.e., the Z-score of LV end-diastolic diameter (LVEDD) was 2 standard deviations above the body surface area (BSA; based on Detroit data) (9) and/or there was cardiac systolic dysfunction with a LVEF of < 45% in the absence of any comorbidities] (4).

Patients with DCM caused by structural heart diseases, myocarditis, infections, arrhythmias, endocrine diseases, neuromuscular diseases, rheumatologic and immunological diseases, nutritional deficiencies, syndromic diseases, conditions leading to ischemia, toxins, and systemic diseases were excluded. Patients with chronic hepatic or kidney diseases were also excluded. Patient survival was evaluated from the time of diagnosis.

Patients were aged 0–15 years. All patients were enrolled after obtaining informed consent from their parents or guardians. Patients were routinely examined using cardiac biomarkers, electrocardiography, and echocardiography. We recorded each patient’s medical and family history, symptoms at presentation, physical signs combined with echocardiography, serum variables, and electrocardiography results. In addition, we tried to complete cardiac magnetic resonance imaging (MRI) when the patients were first hospitalized. But the expensive price and many patients in hemodynamic instability limited the use of cardiac MRI in clinical practice. Only some patients underwent cardiac MRI. Genetic analysis was also performed, including target gene panels and whole-exome sequencing, in 46 patients to identify possible genetic factors. Genetic factors were identified in 16 of 46 patients. The results have been described in a previously published paper (10). The study was approved by the Ethics Committee of Shandong Provincial Hospital and was conducted in accordance with the Declaration of Helsinki.

### Data Collection

Several baseline characteristics, including age, sex, clinical presentation, family history, blood pressure, and history of diuretic use before admission, were recorded at the initial visit. The modified Ross score [class I (0–2), II (3–6), III (7–9), or IV (10–12)] for patients aged ≤ 1 year (11) and the New York Heart Association (NYHA) functional classification for patients aged > 1 year (12) were used to assess cardiac function. Metabolic variables, including hemoglobin, alanine aminotransferase (ALT) (normal ALT concentration 0–50 U/L), N-terminal pro-brain natriuretic peptide (NT-proBNP), creatinine, and serum potassium (normokalemia 3.5–5.0 mmol/L) concentrations, were assessed from blood samples taken on the day of hospitalization by consulting the electronic medical records system. Abnormal potassium was defined as hypokalemia (<3.5 mmol/L) or hyperkalemia (>5.0 mmol/L). Each patient’s weight was recorded, and BSA was calculated. Echocardiography was also performed. We calculated Z-scores for LVEDD to adjust for age and BSA. The degree of mitral regurgitation (MR) and LVEF were also measured. The probability of pulmonary hypertension (PH) was estimated using transthoracic echocardiography (13). PH was defined as a systolic pulmonary arterial pressure of ≥ 30 mmHg. The peak tricuspid regurgitation velocity was measured using continuous-wave Doppler echocardiography, and pulmonary artery systolic pressure was estimated using the simplified Bernoulli equation. Finally, standard 12-lead electrocardiography was recorded at a speed of 25 mm/s and calibrated to 1 mv/cm. Twenty-four-hour Holter monitoring results were recorded if available at onset. We analyzed the presence of arrhythmias, especially severe types of arrythmia, including non-sustained ventricular tachycardia (VT) and atrial tachycardia (AT).

All patients’ medical treatments were also noted. Patients were administered many types of heart failure medication, including diuretics, angiotensin-converting enzyme inhibitors (ACEIs), and β-blockers. Amiodarone was the standard therapy for patients with severe tachyarrhythmias (AT or non-sustained VT). Positive inotropic drugs, including oral digoxin and intravenous drugs like dopamine, dobutamine or milrinone, were administered to some patients with severe disease. Patients with intracardiac thrombosis were administered anticoagulation therapy. We also recorded the use of mechanical ventilation by patients with severe respiratory failure at the time of their first hospitalization.

### Follow-Up

Patient follow-up was performed by telephone interview and by accessing information from cardiologists and/or hospital notes. The main outcome was cardiac mortality, including sudden death (unexpected death in the absence of symptoms or within 1 h of symptom onset in patients with a relatively stable or uneventful course) and heart failure-/VT-related death.

### Statistical Analysis

Statistical analysis was performed using SPSS (version 26.0), R, and GraphPad Prism 8 software. The baseline characteristics of patients are summarized using medians with ranges for continuous variables and percentages for categorical variables. Categorical variables were compared using the chi-square test, and continuous variables were compared using the Kruskal-Wallis test. Patients were divided into non-survival and survival groups based on data from the final follow-up. Survival was estimated using the Kaplan-Meier method. Risk factors were compared using the log-rank test.

Receiver operating characteristic (ROC) curves were used to evaluate the discriminative capacity of a high serum ALT concentration to predict mortality and to analyze the predictive value of the area under the curve (AUC) for mortality. Risk factors for death without heart transplantation were identified with univariate Cox regression modeling. Univariate Cox regression analysis variables included age onset, sex, LVEF, LVEDD Z-score, PH, MR, arrhythmia, cardiac functional classification class III/IV, NT-proBNP concentration, elevated serum ALT concentration, abnormal serum potassium concentration (< 3.0 mmol/L or > 5.0 mmol/L), and use of mechanical ventilation. Hazard ratios (HRs), confidence intervals (CIs), and *p-*values were reported. According to univariate Cox regression results, variables with *P* < 0.1 were selected as candidates for multivariate Cox regression model analysis. A *P-*value of < 0.05 was considered statistically significant.

## Results

### Median and Optimal Cut-Off Values of Aminotransferase Levels

**FIGURE 1:**
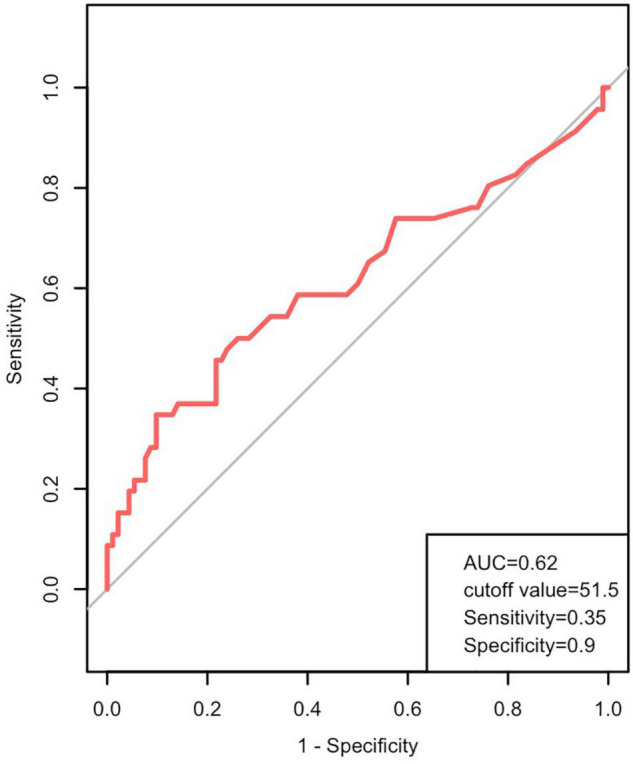
Prognostic value of alanine aminotransferase (ALT) in pediatric patients with DCM. ROC curves showing ALT levels in patients with DCM predicting the cardiac death. AUC, area under the curve; DCM, dilated cardiomyopathy; ROC, receiver operating characteristic.

The median serum ALT level in the total 138 pediatric patients was 25.5 (range: 1–5,558) U/L. As shown in Figure 1, ROC curve analysis was used to assess the prognostic value of ALT for the combined endpoint. The AUC was 0.62, and the optimal ALT cut-off concentration for the endpoint of cardiac death was 51.5 U/L (Youden index 0.25; sensitivity 35%; specificity 90%; *P* < 0.05).

### Baseline Characteristics

Table 1 summarizes the baseline characteristics of 138 patients, including 62 males (45%) and 76 females (55%). The mean age at onset was 10 months (range: 1–180 months). The majority of patients (57.2%) presented before 12 months of age, and 82.8% of patients presented with respiratory symptoms like cough or dyspnea. Other symptoms included failure to thrive in infants and fatigue in older children. No patients were administered diuretics before admission. Advanced heart failure was predicted (cardiac functional classification III/IV) in 121 patients (87.7%). Eight patients (5.8%) had a family history of DCM. Arrhythmias were presented in 54 patients (39.1%), including premature atrial beats, premature ventricular beats, complete left bundle branch block, first-degree atrioventricular block, supraventricular tachycardia, and non-sustained VT, but no cases of brady-arrhythmia. Echocardiographic data showed obvious LV dilatation (median LVEDD Z-score of 6.98) and severe LV dysfunction (median LVEF of 28%). Due to the high cost and a large number of patients with hemodynamic instability, the diagnosis of DCM was confirmed by cardiac MRI in only six patients (4.3%).

**TABLE 1:** Clinical characteristics of pediatric patients with primary DCM in the non-survival and survival groups.

	All (*n* = 138)	Non-survival group (*n* = 46)	Survival group (*n* = 92)	*P*-value
Age, months	10 (1–180)	34 (1.3–168)	8.75 (1–180)	0.002
Age ≤ 12 months, *n* (%)	79 (57.2)	17 (36.9)	62 (67.4)	
>12 months—≤5 years, *n* (%)	29 (21)	13 (28.2)	16 (17.4)	
5 years—≤ 10 years, *n* (%)	12 (8.7)	4 (8.7)	8 (8.7)	
>10 years—≤ 15 years, *n* (%)	18 (13)	12 (26.1)	6 (6.52)	
Respiratory symptoms, *n* (%)	111 (82.8)	38 (82.6)	73 (83)	0.96
Male gender, *n* (%)	62 (44.9)	26 (56.5)	36 (39.1)	0.053
Family history of DCM, *n* (%)	8 (5.8)	3 (6.5)	5 (5.4)	0.797
Laboratory values				
NT-proBNP, pg/ml, (range)	13634.5 (48.17–35,000)	14,255 (48.17–35,000)	12,900 (960–35,000)	0.138
Abnormal potassium, *n* (%)	37 (26.8)	18 (39.1)	19 (20.7)	0.021
Elevated ALT (≥ 51.5 u/L), *n* (%)	25 (18.1)	16 (34.8)	9 (9.8)	0.000
Cardiac functional class III/IV, *n* (%)	121 (87.7)	45 (97.8)	76 (82.6)	0.01
Arrhythmia	54 (39.1)	19 (41.3)	35 (38)	0.711
AT or non-sustained VT, *n* (%)	20 (14.5)	11 (23.9)	9 (9.8)	0.026
Echocardiography parameters				
LVEDD, cm (range)	4.6 (2.48–8.29)	4.8 (2.7–8.29)	4.56 (2.48–7.6)	0.014
LVEDD-*z*-value, (range)	6.98 (2.36–13.75)	6.35 (3.07–12.72)	7.32 (2.36–13.75)	0.09
LVEF, % (range)	28 (13–44)	28.5 (13–40)	27.5 (15–44)	0.858
Left ventricle thrombi, *n* (%)	5 (3.6)	4 (8.7)	1 (1.1)	0.025
PH, *n* (%)	64 (46.4)	26 (56.5)	38 (41.3)	0.091
Moderate or severe MR, *n* (%)	66 (47.8)	23 (50)	43 (46.7)	0.718
Severe MR, *n* (%)	16 (11.6)	5 (10.9)	11 (12)	0.851
Drug therapy after admission, *n* (%)				
Inotropics				
Inotropic drugs intravenously, *n* (%)	54 (39.1)	26 (56.5)	28 (30.4)	0.003
Digoxin, *n* (%)	133 (96.4)	43 (93.5)	90 (97.8)	0.198
Diuretics, *n* (%)	146 (100)	46 (100)	92 (100)	1
ACEIs, *n* (%)	110 (79.7)	27 (58.7)	83 (90.2)	0.000
Beta-blockers, *n* (%)	60 (43.5)	15 (32.6)	45 (48.9)	0.069
Needs for mechanical ventilator, *n* (%)	24 (17.4)	13 (28.3)	11 (12)	0.017
Follow-up time, months, (range)	12 (1 day–88)	2 (1 day–36)	27.5 (1–88)	0.000

*NT-proBNP, N-terminal pro-brain natriuretic peptide; AT, atrial tachycardia; ALT, alanine aminotransferase; VT, ventricular tachycardia; PH, pulmonary hypertension; LVEDD, left ventricular end-diastolic dimension; LVEF, left ventricular ejection fraction; MR, mitral regurgitation; ACEIs, angiotensin-converting enzyme inhibitors.*

**FIGURE 2:**
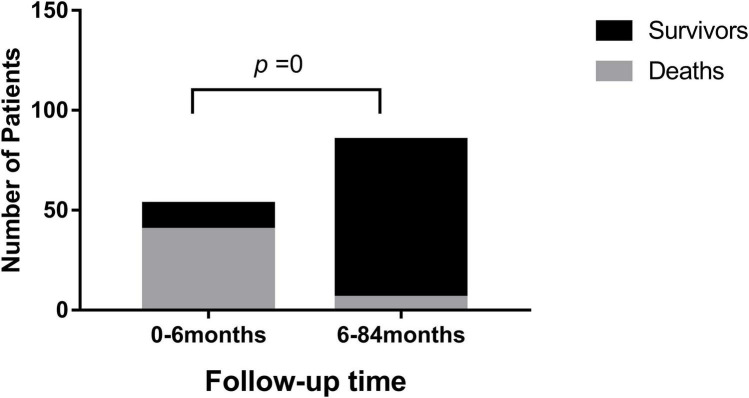
The comparison of mortality according the follow-up time after diagnosis of DCM.

After follow-up (median: 12 months; range: 1 day to 88 months), 46 patients (33%) died as they reached the endpoint. Most deaths (40/46) occurred within 6 months of the DCM diagnosis (*P* = 0) (Figure 2). One patient died from VT, one patient died from sudden cardiac death, and 44 patients died from heart failure. Among the 92 survivors, 50 patients had no symptoms, and LVEF reached above 55%.

Patients were divided into survival and non-survival groups based on this endpoint (Table 1). At the time of diagnosis, there were no significant differences in sex between the two groups (*P* = 0.053). However, the mortality rate was higher in male [*n* = 26 (42%)] than in female patients [*n* = 20 (26.3%)]. More patients showed an elevated ALT concentration (≥51.5 U/L; *n* = 16) than those with low ALT concentration (<51.5 U/L; *n* = 9) in the non-survival group (*P* = 0.000). Children in the non-survival group were older (median age: 34 months) than children in the survival group (median age: 8.75 months) (*P* = 0.002), and had a shorter follow-up time (*P* = 0.000). Patients in the non-survival group were more likely to have an abnormal blood potassium concentration (potassium < 3.0 mmol/L or > 5.0 mmol/L) (*P* = 0.021). No statistical difference was observed in arrhythmia (*P* = 0.711); however, a greater proportion of patients in the non-survival group presented with severe arrhythmia (AT or non-sustained VT) (23.9% vs. 9.8%; *P* = 0.026), and a greater number of patients were classified as cardiac functional class III/IV (*P* = 0.01). More patients underwent mechanical ventilation and intravenous inotropic drug administration in the non-survival group (*P* = 0.003). There was also a statistically significant difference in the incidence of LV thrombus between the two groups [*n* = 4 (8.7%) vs. *n* = 1 (1.1%)] (*P* = 0.025). Except for arrhythmia, there were no statistically significant differences in family history, blood NT-proBNP concentration, and echocardiographic measurements, including LVEF, LVEDD Z-score, PH, and mild or severe MR between the two groups (all *P* > 0.05).

All patients were given medical therapy for heart failure and myocardial remodeling. There were no significant differences in the use of diuretics, oral digoxin, or β-blockers between the two groups, but fewer ACEIs were used in the non-survival group [*n* = 27 (58.7%)] than in the survival group [*n* = 83 (90.2%)] (*P* = 0.000). Intravenous inotropic drugs were more commonly used in the non-survival group [*n* = 26 (56.5%)] than in the survival group [*n* = 28 (30.4%)] (*P* = 0.003) due to the increased severity of the condition in patients who did not survive. A greater number of patients [*n* = 13 (28.3%)] with respiratory failure required mechanical ventilation in the non-survival group than in the survival group [*n* = 11 (12%)] (*P* = 0.017). Because amiodarone was effective for non-sustained VT and no brady-arrhythmia was observed, implantable cardioverter defibrillator or pacemaker implantation was unnecessary.

### Survival Analysis

**FIGURE 3:**
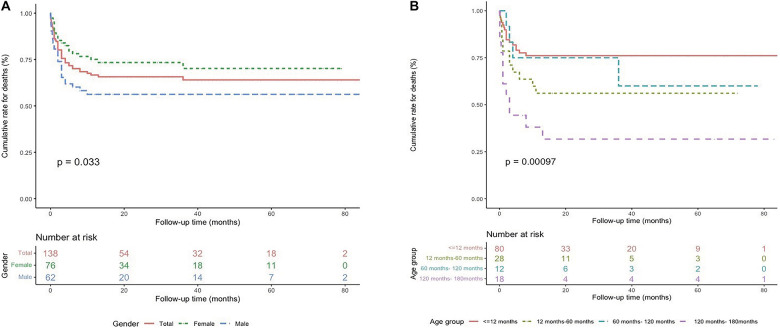
Survival rates from Kaplan–Meier estimates among the study population by sex **(A)** and age at diagnosis **(B)**.

The Kaplan–Meier analysis indicated a higher probability of mortality in male patients (*P* = 0.033), and patients with a particular age at onset (*P* = 0.001) (Figure 3), suggesting a complex relationship between age-onset and mortality, with a higher mortality rate in patients aged 10–15 years or 12 months–5 years at diagnosis.

The cox univariable regression analysis showed that predictors of death included age at diagnosis (HR 1.008; 95% CI 1.003–1.013; *P* = 0.001), male sex (HR 1.855; 95% CI 1.035–3.325; *P* = 0.038), cardiac functional class III/IV (HR 7.469; 95% CI 1.029–54.199; *P* = 0.047), presence of severe arrhythmia (AT or non-sustained VT; HR 2.376; 95% CI 1.205–4.685; *P* = 0.013), an abnormal potassium concentration (HR 2.087; 95% CI 1.154–3.774; *P* = 0.015), an elevated ALT concentration (HR 3.776; 95% CI 2.050–6.954; *P* = 0.000), intravenous inotropic drug use (HR 2.614; 95% CI 1.456–4.692; *P* = 0.001), and the requirement for mechanical ventilation (HR 2.796; 95% CI 1.468–5.325; *P* = 0.002) (Table 2).

**TABLE 2:** Predictors of death by the cox regression analysis.

Variables	Univariate analysis			Multivariate analysis		
	HR	95% CI	*P*-value	HR	95% CI	*P*-value
Sex, months	1.855	1.035–3.325	0.038	1.647	0.869–3.121	0.126
Age, months	1.008	1.003–1.013	0.001			
Age ≤ 12 months	1		0.003	1		0.057
>12 months— ≤ 5 years	2.024	0.975–4.203	0.059	2.799	1.160–6.758	0.022
5 years–≤ 10 years	1.349	0.456–3.987	0.588	2.347	0.654–8.421	0.191
> 10 years—≤ 15 years	3.981	1.915–8.276	0.000	3.617	1.336–9.788	0.011
Cardiac functional class III/IV, *n* (%)	7.469	1.029–54.199	0.047	4.255	0.562–32.246	0.161
Family histories	1.019	0.316–3.287	0.975			
NT-proBNP, pg/ml	1.878	0.994–3.550	0.052			
Abnormal potassium	2.087	1.154–3.774	0.015	1.873	0.947–3.702	0.071
Elevated ALT	3.776	2.050–6.954	0.000	2.219	1.06–4.574	0.031
LVEDD-*z*-value	0.917	0.823–1.021	0.114			
LVEF	0.356	0.004–28.581	0.645			
PH	1.653	0.922–2.962	0.091			
Moderate or severe MR	1.045	0.586–1.863	0.881			
AT or non-stained VT	2.376	1.205–4.685	0.013	1.736	0.781–3.86	0.176
Inotropic drugs intravenously	2.614	1.456–4.692	0.001	0.951	0.461–1.962	0.892
Mechanical ventilator	2.796	1.468–5.325	0.002	4.223	1.763–10.114	0.001

*NT-proBNP, N-terminal pro-brain natriuretic peptide; AT, atrial tachycardia; ALT, alanine aminotransferase; VT, ventricular tachycardia; PH, pulmonary hypertension; LVEDD, left ventricular end-diastolic dimension; LVEF, left ventricular ejection fraction; MR, mitral regurgitation; HR, hazard ratio; CI, confidence interval. NT-proBNP concentration was analyzed after logarithmic transformation; elevated serum ALT concentration: ≥ 51.5 U/L; abnormal serum potassium concentration: < 3.0 mmol/L or > 5.0 mmol/L.*

The cox multivariable regression analysis showed that mortality predictors included an age at diagnosis of 12 months to 5 years (HR 2.799; 95% CI 1.160–6.758; *P* = 0.022) or 10–15 years (HR 3.617; 95% CI 1.336–9.788; *P* = 0.011), an elevated ALT concentration (≥51.5 U/L) (HR 2.219; 95% CI 1.06–4.574; *P* = 0.031), and the need for mechanical ventilation (HR 4.223; 95% CI 1.763–10.114; *P* = 0.001) (Table 2).

## Discussion

DCM, one important cause of heart transplantation in children (14), can cause sudden cardiac death in pediatric patients (15). The present study aimed to identify the clinical characteristics of pediatric patients with primary DCM without heart transplantation in an Asian population and the risk factors that predict mortality. We showed that the mortality rate among the 138 patients was 33%, which is similar to previous reports (6, 16).

Multiple risk factors like respiratory symptoms, sex distribution, family history, arrhythmia, LVEDD Z-score, and LVEF were investigated in this study, but no statistical differences were shown between the survival and non-survival groups (Table 1). A family history of DCM was found in 8 patients but was not determined as a predictor of mortality by cox univariate analysis (Table 2).

Consistent with a previous study (17), the highest mortality rate occurred in the first few months after diagnosis as shown in Figure 2. All of the deaths occurred within 3 years after diagnosis (Table 1), after which there was a relatively stable period. Among the 92 survivors, 50 had no symptoms, and LVEF reached > 55%. A longer follow-up may be necessary because some children have died or underwent heart transplantation for recurrent heart failure (17), which can be reached by future investigations.

DCM is known to more commonly affect men than women (18, 19). In our study, males accounted for 45% of the whole patients, but the Kaplan–Meier curve showed higher mortality in male patients (Figure 3A). However, sex was not a determining risk factor for mortality after the cox multivariable analysis (Table 2).

Previous studies have shown the correlation between age at diagnosis and the death or transplantation in pediatric patients with DCM but the results are inconsistent (20–23). Our Kaplan–Meier cumulative survival curves identified two periods (i.e., patients diagnosed at 12 months to 5 years of age and at 10–15 years of age) having a higher risk of death (Figure 3B), and the two age ranges at diagnosis correlated with an increased hazard of death (Table 2). Importantly, most heart failure deaths (40/46) occurred within the first 6 months after diagnosis (Figure 2), which is consistent with a previous study (24). Thus, treatment methods for DCM, including medical treatment, LV-assist devices, and heart transplantation, should be carefully designed at the initial presentation.

Echocardiography parameters, including LVEDD, age, LV fractional shortening Z-score, and the degree of MR are believed to play a role in predicting death, but the study results are conflicting (25). Here, we analyzed the risk factors for cardiac death by using cox multivariable regression analysis (Table 2), and found that LVEDD, LVEF, degree of MR, and PH were not associated with increased mortality, which is in accordance with previous findings (26).

In line with the known relationship between congestion and hypoperfusion of the liver and heart failure (27), liver dysfunction, such as ALT and/or aspartate aminotransferase elevation, are prevalent in patients with DCM (28–30). It is also known that patients with chronic heart failure with severe hepatic dysfunction have poor outcomes (31–33). However, others have found that aminotransferase concentrations are not associated with worse clinical outcomes (34, 35).

In this cohort, distinct from other studies, we used ROC curves to evaluate the risk of mortality using the serum ALT concentration. We determined that the optimal ALT cut-off concentration for the endpoint of cardiac death was 51.5 U/L. This concentration is a little above the normal concentration range (0–50 U/L). Through both univariate and multivariate cox regression analyses, we identified the role of risk factors in predicting cardiac death among pediatric patients with DCM. This finding further confirmed that circulatory failure could cause congestive hepatopathy and/or acute cardiogenic liver injury.

Hyperkalemia and hypokalemia are both associated with an increased risk of mortality in patients with heart failure (36). The potassium concentration often fluctuates based on diet, medication, and changes in renal function. In this study, patients with severe disease in the non-survival group more often had cardiac functional class III/IV disease. The severity of the disease led to poor intake of diet and metabolic derangements. Correspondingly, more patients with an abnormal potassium concentration were observed in the non-survival group than in the survival group. However, the cox multivariate analysis showed that an abnormal potassium concentration could not predict cardiac death independently.

Patients with severe DCM may develop respiratory failure and requires mechanical ventilation to help to increase intrathoracic pressure, reduce right ventricular and LV preload and LV afterload, improve dyspnea, and augment oxygenation (37). Mechanical ventilation also has favorable effects on hemodynamics in patients with heart failure (38, 39). We found that 24 patients (17.4%) required mechanical ventilation, and 11 patients survived while others died. The cox multivariable regression analysis determined the use of mechanical ventilation as an independent prognostic indicator for the risk of cardiac death in DCM pediatric patients.

In this study, patients were given diuretics (100%), ACEIs (79.7%), oral digoxin (96.4%), and inotropic drugs intravenously (39.1%), as well as β-blockers (43.5%) at the first admission as recommended by guidelines. No significant statistical differences existed in the use of diuretics, oral digoxin, and β-blockers between the two groups, but fewer ACEIs were used in the non-survival group than in the survival group. The medication with ACEIs was not applicable in some cases due to severe heart failure, unstable hemodynamics, low blood pressure, side effects (cough and hypotension), or the death that occurred at the first admission. However, all patients used ACEIs when re-admitted in a stable status. Because of a lack of experience applying ARBs in cases of intolerance to ACEIs in children, no ARBs were used. Also, more intravenous inotropic drugs were used in the non-survival group, which reflected the severity of the condition at presentation.

The oral inotrope and neurohormonal modulator—digoxin is a commonly used drug in treating heart failure and DCM (40). Digoxin can reduce hospitalization and the possibility of all-cause mortality and improve exercise tolerance (41). Most patients in our study underwent digoxin treatment, but no difference was observed between the two groups.

The univariate analysis showed that NT-proBNP concentration, cardiac functional class III/IV, abnormal potassium concentration, presence of AT, use of intravenous inotropic drugs, and presence of severe arrhythmia (AT or non-sustained VT) were related to cardiac mortality. However, in the multivariate analysis, none of these factors were decisive.

The presence of LV thrombus was different between the two groups, but the numbers were too small to reach significance. The requirement for mechanical ventilation and an elevated ALT concentration were important indicators of cardiac death. Therefore, heart transplantation should be considered more readily in patients with multiple-organ failure.

## Limitations

First, the study cohort was small, and patients were enrolled at a single center, reflecting selection bias. Especially there were too few subjects in group of 5–10 years, this would lead to the instability of the model. Conclusions might not be so valid. Second, the absence of routine endomyocardial biopsy may have led to an underestimation of the population with chronic cardiac inflammation and an overestimation of the incidence of primary DCM. Third, serum parameters were measured at only one time point; we did not evaluate the data as clinical features worsened. Fourth, genetic testing and cardiac MRI were not routinely available throughout the study period; therefore, some specific etiologies may have been missed. Because of the limited number of patients who underwent a genetic analysis, we did not include genetic factors in our model. Fifth, the follow-up period for some patients was not long enough to draw firm conclusions, despite there being consistent clinical work-up and follow-up for most patients. Finally, we did not identify medication compliance and detailed anti-heart failure medications. Moreover, we did not examine the impact of medical therapy on heart failure symptoms and outcomes. Thus, more evidence is needed to draw firm conclusions about whether outcomes in children with DCM are modified by various medical therapies, including the use of LV-assist devices and medications.

## Conclusion

In summary, this study investigated the clinical characteristics and outcomes of pediatric patients with primary DCM without heart transplantation in China. Our data show that there is a high mortality rate in Chinese pediatric patients with DCM who do not undergo heart transplantation. The period with the greatest risk of cardiac death among pediatric patients with DCM was the first 6 months after diagnosis. Early mortality was common in patients, but the clinical status of long-term survivors was often good. Survival outcomes were worse for subjects diagnosed at the age of 12 months to 5 years of age, those diagnosed at 10–15 years of age, patients with an elevated ALT concentration, and patients who needed mechanical ventilation at the time of presentation. More precise risk stratification and personalized therapy may considerably improve outcomes for these patients.

## Data Availability Statement

The raw data supporting the conclusions of this article will be made available by the authors, without undue reservation.

## Ethics Statement

The studies involving human participants were reviewed and approved by the Ethics Committee of Shandong Provincial Hospital. Written informed consent to participate in this study was provided by the participants’ legal guardian/next of kin. Written informed consent was obtained from the individual(s), and minor(s)’ legal guardian/next of kin, for the publication of any potentially identifiable images or data included in this article.

## Author Contributions

YW and BH collected patient data and prepared the manuscript. YF, XY, JL, and JW contributed to the clinical understanding of the case and revision of the manuscript. YY, HY, LZ, and JZ analyzed, interpreted the genetic data, and surveyed the literature relevant to the mutations. All authors contributed to the article and approved the submitted version.

## Conflict of Interest

The authors declare that the research was conducted in the absence of any commercial or financial relationships that could be construed as a potential conflict of interest.

## Publisher’s Note

All claims expressed in this article are solely those of the authors and do not necessarily represent those of their affiliated organizations, or those of the publisher, the editors and the reviewers. Any product that may be evaluated in this article, or claim that may be made by its manufacturer, is not guaranteed or endorsed by the publisher.
